# S232. ALPHA7 NICOTINIC RECEPTOR AGONISTS REVERSE THE HYPERDOPAMINERGIC STATE IN THE MAM MODEL OF SCHIZOPHRENIA

**DOI:** 10.1093/schbul/sby018.1019

**Published:** 2018-04-01

**Authors:** Gilda Neves, Anthony Grace

**Affiliations:** University of Pittsburgh

## Abstract

**Background:**

Most investigations into the pharmacology of schizophrenia have revolved around dopaminergic and glutamatergic neurotransmission; however, one neurotransmitter that has not received adequate attention is the cholinergic system. Indeed, several post-mortem, genetic and epidemiologic studies link specifically the alpha7 nicotinic receptor (nAChR) to schizophrenia, and the potential use of alpha7 modulators as a treatment strategy is an active field of research. Nevertheless, studies to date have been limited to normal animals rather than on a validated neurodevelopmental model of schizophrenia. Moreover, knowledge about the differential impact of orthosteric and allosteric modulators in vivo is lacking. Thus, we investigated the effects of alpha7 nAChR modulation on dopamine (DA) neuron activity in the ventral tegmental area (VTA) in the methylazoxymethanol acetate (MAM) animal model of schizophrenia.

**Methods:**

All experimental procedures were conducted according to NIH guidelines and were approved by University of Pittsburgh Institutional Animal Care and Use Committee. Sprague-Dawley pregnant dams were treated with MAM or saline on gestational day 17. Recordings of VTA dopamine neuron activity was performed on the male offspring at adulthood. The effects of four different drugs were evaluated: PNU282987 (full agonist), SSR180711 (partial agonist) NS1738 (type I positive allosteric modulator - PAM) and PNU120596 (PAM type II).

**Results:**

Intravenous administration of alpha7 selective ligands did not induce a major change in the firing profile of spontaneously active DA neurons when dosed during dopamine neuron recording. PNU120596 increased in the number of active DA neurons found in the VTA of normal rats, their mean firing rate and percentage of spikes in bursts. In contrast, the full agonist PNU282987 and the partial agonist SSR180711 reduced the hyperdopaminergic tone in MAM rats, with a more prominent decrease in the number of DA neurons recorded in the lateral VTA. In order to investigate the drug site of action, both PNU282987 and SSR1800711 were infused into the ventral hippocampus (vHipp) and basolateral amygdala (BLA). After vHipp infusion, the alpha7 nAChR agonists significantly decreased the number of active DA neurons in MAM rats, with no significant impact in control rats. Once more, the effects were more robust in the lateral VTA. In contrast, the same drugs when infused directly into the BLA increased the number of spontaneously active DA neurons in the VTA of normal rats, but not in the MAM model.

**Discussion:**

In summary, our results show that alpha7 nAChR positive modulators can affect midbrain dopaminergic neuronal activity in vivo in a state-dependent manner. Interestingly, alpha7 nAChR agonists counterbalanced the hyperdopaminergic state of MAM rats and this effect is partially mediated by their action in the vHipp. This effect is consistent with the potential use of alpha7 nAChR agonists for schizophrenia treatment and fits the current search for drugs able to control dopaminergic function acting in structures upstream from the dopamine receptor. The predominant inhibition of the lateral VTA points to a lower propensity to produce unwanted side effects in comparison to current employed antipsychotic agents. Our data show that drug effects can vary according to the basal level of activity of specific brain circuits and highlights the importance of using appropriated animal models to make inferences about potential therapeutic use of new neuropsychiatric drug candidates.

